# Tyrosine Kinase Inhibitors Improved Survival of Critically Ill EGFR-Mutant Lung Cancer Patients Undergoing Mechanical Ventilation

**DOI:** 10.3390/biomedicines9101416

**Published:** 2021-10-08

**Authors:** I-Hsien Lee, Ching-Yao Yang, Jin-Yuan Shih, Chong-Jen Yu

**Affiliations:** 1 Department of Emergency and Critical Care Medicine, Fu-Jen Catholic University Hospital, New Taipei City 24308, Taiwan; qqcu111@gmail.com; 2 Division of Thoracic Medicine, Department of Internal Medicine, National Taiwan University Hospital, Taipei 10225, Taiwan; jyshih@ntu.edu.tw (J.-Y.S.); jefferycjyu@ntu.edu.tw (C.-J.Y.)

**Keywords:** EGFR, lung cancer, critical care, mechanical ventilation, tyrosine kinase inhibitor

## Abstract

Background: Respiratory failure requiring mechanical ventilation is the major reason for lung cancer patients being admitted to the intensive care unit (ICU). Though molecular targeted therapies, especially epidermal growth factor receptor (EGFR)-tyrosine kinase inhibitors (TKIs), have largely improved the survival of oncogene-driven lung cancer patients, few studies have focused on the performance of TKI in such settings. Materials and Methods: This was a retrospective cohort study enrolling non-small cell lung cancer (NSCLC) patients who harbored sensitizing EGFR mutation and had received EGFR-TKIs as first-line cancer therapy in the ICU with mechanical ventilator use. The primary outcome was the 28-day ICU survival rate, and secondary outcomes were the rate of successful weaning from the ventilator and overall survival. Results: A total of 35 patients were included. The 28-day ICU survival rate was 77%, and the median overall survival was 67 days. Multivariate logistic regression revealed that shock status was associated with a lower 28-day ICU survival rate independently (odds ratio (OR) 0.017, 95% confidence interval (CI), 0.000–0.629, *p* = 0.027), and that L858R mutation (L858R compared with exon 19 deletion, OR, 0.014, 95% CI 0.000–0.450, *p* = 0.016) and comorbidities of diabetes mellitus (DM) (OR, 0.032, 95% CI, 0.000–0.416, *p* = 0.014)) were independently predictive of weaning failure. The successful weaning rate was 43%, and the median of ventilator-dependent duration was 22 days (IQR, 12–29). Conclusions: For EGFR mutant lung cancer patients suffering from respiratory failure and undergoing mechanical ventilation, TKI may still be useful, especially in those with EGFR del19 mutation or without shock and DM comorbidity.

## 1. Introduction

Lung cancer patients account for 8% of all intensive care unit (ICU) admissions of patients with malignancies and 27% of those with solid cancer [1,2]. However, lung cancer patients have experienced worse ICU outcomes than those with other solid cancers. Data from the Surveillance, Epidemiology, and End Results (SEER) Medicare registry (1992 to 2007, N = 49,373) revealed that 65% of patients with lung cancer died within 6 months after ICU admission [3]. A recent large multi-center retrospective cohort study reported modest improvements in lung cancer patient survival—they found that 449 patients admitted to 22 ICUs in Europe and Latin America had 6-month survival rates between 40% and 50% [4]. Patients with a non-progressive malignancy and good performance status (PS score ≤ 2) [4] had a better prognosis. Although the outcomes of patients with lung cancer admitted to the ICU in different studies varied, overall ICU mortality was around 50%. The use of mechanical ventilation (MV) for lung cancer patients who developed acute respiratory failure was associated with a mortality rate of over 70% [3,5,6]. Treating patients with advanced non-small-cell lung cancer (NSCLC) using chemotherapy in the ICU is controversial because a PS score >2 is considered to be a contraindication for chemotherapy administration, and NSCLC is usually less sensitive to chemotherapeutic drugs [7]. By the mid-2000s, ICU admission for life-threatening events was still widely viewed as unlikely to benefit these patients, particularly when ventilator support is needed [8].

However, in the 21st century, targeted therapy has dramatically changed the management of NSCLC. In 2009, a landmark trial described a “Lazarus” response in NSCLC patients with a PS of 34—a dramatic improvement in PS was found in 70% of patients who harbored an EGFR mutation [9,10]. Tumors that harbor EGFR mutations can exhibit dramatic responses to an EGFR-tyrosine-kinase inhibitor (TKI), such as gefitinib, erlotinib, afatinib, or osimertinib [11,12,13,14]. However, there is limited evidence suggesting the use of TKI in EGFR-mutant lung cancer patients who suffer from respiratory failure and need ICU admission. A few case series exist regarding the use of targeted therapy for patients with NSCLC in the ICU [6,15,16,17,18,19]. Besides targeted therapies, immune checkpoint inhibitors have also refined the paradigm of lung cancer treatment in the past decade, especially in patients with high programmed death-ligand 1 (PD-L1) expression [20,21]. Unlike chemotherapy or small molecule inhibitors, immunotherapy further improved long-term survival in a subset of patients, making a long tail in the overall survival curve [22]. However, the effectiveness of immunotherapy is probably limited in patients suffering from critical illness, who are mostly in an immunocompromised status [23,24,25].

Since targeted therapy has better efficacy and fewer treatment-related side effects, namely, it is more tolerable for patients even in a critical status, compared to cytotoxic chemotherapy, treating ICU patients with EGFR-TKIs if the sensitizing mutation is identified could be a reasonable approach. In this study, we aimed to analyze the performance of TKI with lung cancer patients admitted to the ICU due to respiratory failure and who required MV, and of whom all had an available EGFR mutation status.

## 2. Materials and Methods

### 2.1. Study Design and Patient Population

This was a single-center retrospective study, conducted from 2010 to 2018 at National Taiwan University Hospital, which has 5 medical ICUs and a total of 49 beds. The inclusion criteria were as follows: advanced NSCLC, available EGFR mutation status, admission to the ICU with respiratory failure and undergoing MV, use of EGFR-TKIs during ICU hospitalization, and no tumor progression if the EGFR-TKI was given before ICU admission. The study was approved by the Research Ethics Committee of our hospital (201802015RINB).

### 2.2. Data Collection and Outcome

After enrollment, demographics and baseline characteristics such as age, sex, co-morbidity, ICU admission diagnosis, and illness severity upon ICU admission (APACHE II score) were recorded for all patients. Other clinical data including cancer stage, lung cancer histologic type (NSCLC), molecular status, and metastases sites were recorded. The primary reasons for ICU admission were categorized as pulmonary, septic shock, cardiac, or neurological. The treatments given in the ICU, including MV, vasopressor, dialysis, and do not resuscitate (DNR) orders, were recorded. The types and duration of EGFR-TKIs for lung cancer treatment were also recorded. The primary end point was 28th day survival in the ICU. Other secondary end points included discharge status from the ICU, 28th day mortality in the hospital, discharge status from the hospital, and MV weaning results.

### 2.3. Detection of EGFR Mutations

The preservation and preparation for the biopsied tumors were all formalin-fixed paraffin-embedded (FFPE) specimens. Mutational analysis of EGFR testing was performed in an ISO 15189-certificated central lab. Briefly, genomic DNA was extracted using the QIAmp DNA Minikit (QIAGEN, Redwood City, CA, USA), and the mutations were detected by the MassARRAY system (Agena, San Diego, CA, USA), based on the user manual. Extracted DNA was subjected to serial biochemical reactions, including 40 cycles of PCR, shrimp alkaline phosphatase (SAP) treatment, and 200 cycles of a signal nucleotide extension reaction. After cleaning using SpectroCLEAN resin, samples were loaded onto the matrix of a SpectroCHIP by Nanodispenser (Matrix), and then analyzed using Bruker Autoflex MALDI-TOF MS. Data were collected and analyzed using Typer4 software (Agena Bioscience, San Diego, CA, USA).

### 2.4. Statistical Analysis

Baseline demographics were compared between groups. All categorical variables were analyzed using Pearson’s χ2 tests, except where a small sample size (<5) required the use of Fisher’s exact test. Continuous variables were analyzed using the Wilcoxon rank-sum test. Univariate and multivariate logistic regression was performed for 28-day ICU survival and weaning outcome. The odds ratios (ORs), 95% confidence intervals (CIs), and *p*-values were reported. After univariate analysis, the factors with *p*-value < 0.1 and with clinical importance were enrolled into multivariate analysis. ICU and days of MV use were compared by log-rank test and were plotted using Kaplan–Meier methods by the group of significant predictors. Statistical significance was set at a 2-sided *p* < 0.05. All analyses were performed using STATA version 15.0.

## 3. Results

### 3.1. Patient Characteristics

**Figure 1:**
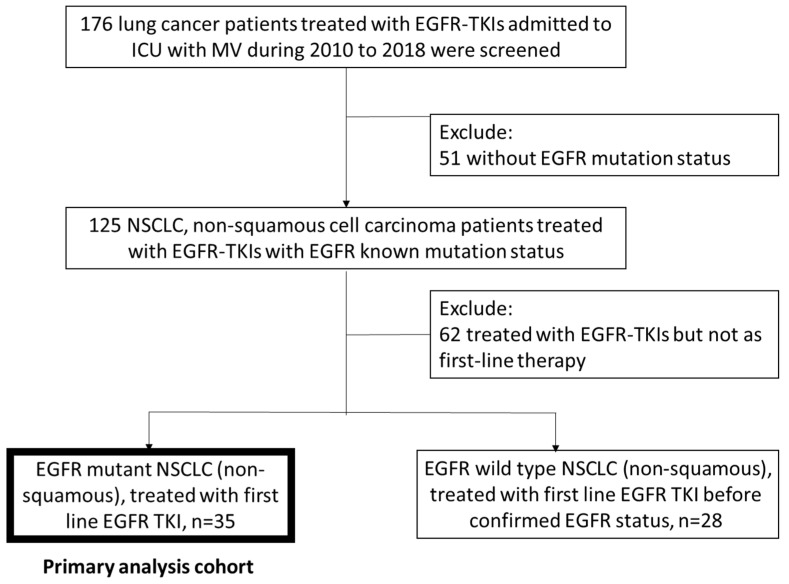
Consortium diagram of our study. Acronyms: EGFR = epidermal growth factor receptor, ICU= intensive care unit, MV = mechanical ventilation, NSCLC = non-small cell lung cancer, TKI = tyrosine kinase inhibitor.

From 2010 to 2018, 176 patients admitted to the ICU with MV use and the diagnosis of NSCLC, and who were treated with EGFR-TKI, were enrolled. Fifty-one patients were excluded due to a lack of documentation of EGFR mutation status. Another 62 patients were excluded because of previous use of chemotherapy or other TKIs. Sixty-three patients who received EGFR-TKIs as first-line therapy for lung cancer, and among whom 35 harbored a sensitizing EGFR mutation were included. The median age of the patients was 73 years, 66% of the patients were female, and 77% were never-smokers. In terms of comorbidities, 9% of the patients had coronary artery disease or heart failure, and 17% had COPD. The major reason for ICU admission and MV use was pneumonia (80%). Most of the patients (34 of 35, 97%) were diagnosed with adenocarcinoma, and 1 patient had sarcomatoid carcinoma. Ninety-seven percent of the patients had stage 4 lung cancer, according to the American Joint Committee on Cancer 7th edition, and 23% had brain metastases. The mutation subtypes of the patients who had a sensitizing EGFR mutation were as follows: L858R: 15 (42.8%), exon 19 deletion: 14 (40%), and uncommon mutation: 6 (17.1%). Mean APACHE II score of the patients was 25 (22–28) (Table 1). The CONSORT diagram is shown in Figure 1.

### 3.2. Clinical Outcomes in the ICU

Most of the patients were treated with a first- or second-generation EGFR-TKI (gefitinib: 22; erlotinib: 11; and afatinib: 1). Only one patient received osimertinib treatment in the ICU. The median duration for the use of EGFR-TKIs in the ICU was 17 days for patients with a sensitizing EGFR mutation.

**Figure 2:**
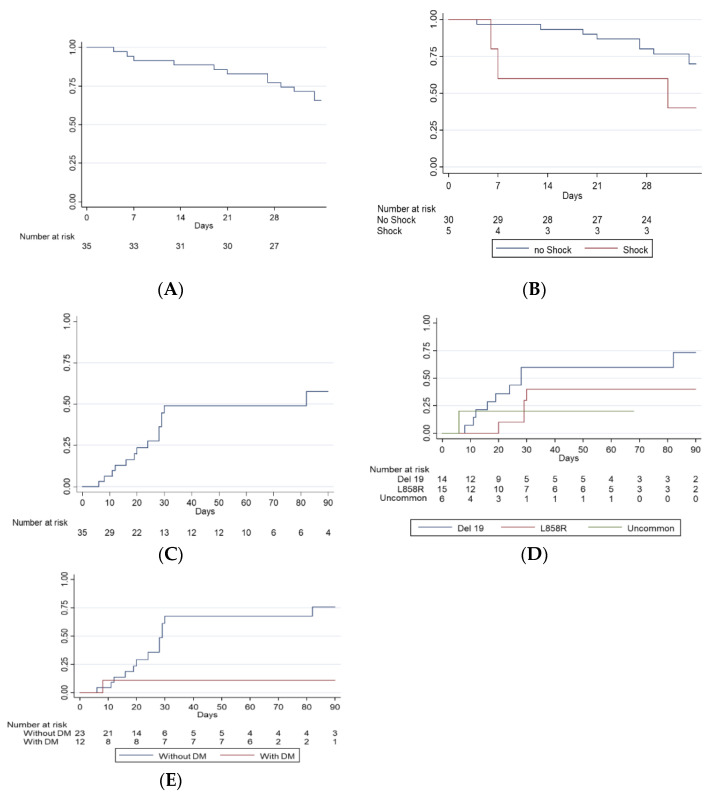
Survival and rate of successful weaning from mechanical ventilation of lung cancer patients receiving EGFR-TKIs in the ICU. (**A**) 28-day ICU survival. (**B**) Kaplan–Meier plot of survival in group with shock or not. (**C**) Cumulative incidence of patients with successful weaning from mechanical ventilators. (**D**) Cumulative incidence of successful weaning in patients with different EGFR mutation. (**E**) Cumulative incidence of successful weaning in patients with or without DM. Acronyms: EGFR = epidermal growth factor receptor, ICU = intensive care unit, TKI = tyrosine kinase inhibitor, DM = diabetes mellitus.

The 28-day ICU survival rate was 77%, and the median survival time was 67 days. Multivariate logistic regression revealed that shock status at ICU admission effectively predicted 28-day ICU survival (OR 0.017, 95% CI, 0.000–0.629, *p* = 0.027) (Table 2). The 28-day ICU survival curve is shown in Figure 2A. The log rank test showed significantly better 28-day in patients without shock, with a *p* value < 0.001 (Figure 2B).

In addition, 43% of the patients were successfully weaned from MV, and the median days with MV use was 22 (IQR = 12–29) days (Figure 2C). The cumulative incidence of successful weaning rate was higher among the patients harboring EGFR deletion 19 mutation than those with L858R or other uncommon mutations, with a log-rank *p* value of 0.016 (Figure 2D); it was also higher in the patient without diabetes mellitus (DM) (log-rank *p* value < 0.001, Figure 2E). Multivariate logistic regression yielded that L858R (compared to Deletion 19, OR 0.014, 95% CI 0.000–0.450, *p* = 0.016) and DM (OR 0.014, 95% CI 0.000–0.416, *p* = 0.014) were independently predictive of weaning failure (Table 3).

Otherwise, there were 28 mechanically ventilated EGFR wild type lung cancer patients who also received EGFR TKI in ICU during our study period. Most of them stopped EGFR TKI treatments after the wild-type status had been confirmed, and the median duration of EGFR TKI of them was 8 days. The demographic data of these patients are shown in Supplementary Table S1. Compared to EGFR mutant cases, EGFR wild type patients had shorter 28-day, 90-day and overall survival (Supplementary Figure S1 and Table S2), and the successful weaning rate was only 25% (7 of 28).

Regarding TKI efficacy, there were only 16 of 35 patients receiving follow-up chest computed tomography (CT) to evaluate treatment response, though most of the CT scans were performed after ICU discharge. In EGFR mutant cases with evaluable CT results (*n* = 16), all showed partial response to EGFR TKI, but only 12 of 16 were successfully weaned from mechanical ventilation. In EGFR mutant cases without CT studies (*n* = 19), 3 of 19 showed radiologically improvements in chest radiography, and all 3 patients were weaned from mechanical ventilation. The results are summarized in Table 4.

**Figure 3:**
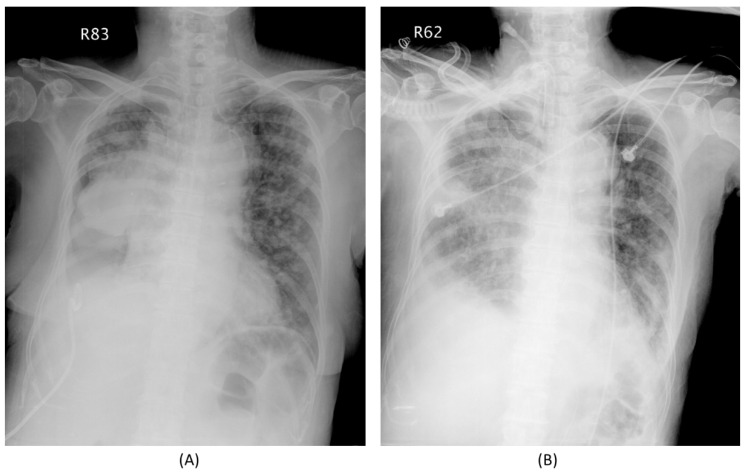
Case of a patient who experienced a dramatic response to EGFR-TKI during ICU treatment. This 72-year-old woman had right lung adenocarcinoma, cT4N3M1c, stage IVB, with malignant pleural effusion, lung-to-lung, liver, and adrenal metastasis. She suffered from an episode of pneumonia and hypercapnic respiratory failure, and was admitted to the ICU for intensive care. The plain film before (**A**) and 2 weeks after gefitinib (**B**) are shown. The patient finally was weaned successfully from the mechanical ventilator and discharged home with tracheostomy under ambient air. Acronyms: EGFR = epidermal growth factor receptor, ICU = intensive care unit, TKI = tyrosine kinase inhibitor.

We present an EGFR-mutant case that received EGFR-TKI treatment after ICU admission and subsequently experienced a remarkable response (Figure 3). Briefly, this 72-year-old woman presented with respiratory failure due to tumor obstruction of the right main bronchus and total collapse of the right lung. After 14-day gefitinib use, the right main bronchus obstruction was resolved, and the patient was successfully weaned from MV.

### 3.3. Treatment Toxicity in the ICU

Interstitial pneumonitis developed in two patients (6%), of whom one used gefitinib and one used erlotinib. TKI was withheld, but one patient (treated with erlotinib) still died despite systemic steroid treatments. Other adverse events, including diarrhea (2 of 35, 6%), hepatitis (1 of 35, 3%), and skin toxicity (4 of 35, 11%), occurred, but did not exceed grade 3; thus, TKI treatment was kept without interruption.

### 3.4. Patient Deposition after ICU Discharge

Of the 27 patients who survived up to the 28th day after ICU admission, 18 were successfully discharged from the hospital. The median length of stay was 21 (interquartile range: 15–31) days in the ICU and 42 (interquartile range: 33–68) days in the hospital. In addition, eight patients returned home without MV use, one returned home with MV use, one was transferred to a long-term respiratory care unit with MV use, and one patient was transferred to a nursing home.

## 4. Discussion

For lung cancer patients suffering from respiratory failure and admitted to the ICU, administration of an effective anti-cancer therapy, in addition to critical care management, is crucial. Our study showed that TKIs could prolong ICU survival in EGFR-driven lung cancer, even for those patients with a critical illness requiring MV. Patients who harbor EGFR exon 19 deletion, who were hemodynamically stable, and who had no DM comorbidity may benefit more from EGFR TKI. To our knowledge, this is the largest cohort to date that substantiates the benefit of EGFR-TKI use for lung cancer patients in such a setting.

In the past, the benefits of medical ICU admission and MV for critically ill lung cancer patients were held in doubt [3,26,27,28,29]. The overall ICU and in-hospital mortality rates in our study group were only 23% and 51%, respectively. Mortality in our study group was less than that of previous studies on the survival of lung cancer patients admitted to the medical ICU with MV use, in which ICU mortality ranged from 40 to 60% and in-hospital mortality ranged from 50 to 80% [3,26,27,28,29,30,31]. As reported in studies prior to 2010, best supportive care was the main treatment strategy for lung cancer patients [3]. In our study, all patients who received EGFR-TKI therapy were documented to harbor a sensitizing EGFR mutation. The better survival in our study was probably due to the use of EGFR-TKIs, and the additional benefits in the del19 subgroup were also consistent with the results in clinical trials [11,32]. Otherwise, DM is another risk factor found in our study to predict weaning failure. Though plenty of researchers have demonstrated the disadvantage of DM in critically ill patients [33], the specific impact on weaning is still undetermined [34] and needs larger studies to clarify.

With the advent of the era of TKIs, treatment for lung cancer patients with a poor performance status changed [9]. Several small case series reported the efficacy of TKIs in lung cancer patients admitted to the medical ICU. Some studies evaluated the efficacy of EGFR-TKIs for NSCLC patients admitted to the ICU with MV use [6]. Hsia et al. reported a study that enrolled 83 patients, of whom only 23 were treated with EGFR-TKIs in 2014. The use of EGFR-TKIs made no difference in hospital mortality (68% vs. 61%, *p* = 0.81) and weaning rate (18% vs. 22%, *p* = 0.81) in the standard care and TKI groups. Instead, the SAPS and SOFA scores were significant predictors of weaning outcome. Toffart et al. (2015) reported that the use of TKIs had no impact on early mortality, but improved survival for those at a late phase (28 days after ICU admission) only [35]. These previous results suggested that weaning and mortality were determined by the severity of the critical illness. None of them demonstrated the independent prognostic role of EGFR mutation in the setting of TKI treatment for lung cancer patients admitted to the ICU due to respiratory failure. Kerrigan et al. [17] and Chen et al. [36] also reported the use of TKIs with critically ill lung cancer patients, but the case number of patients with a documented mutation status in the two studies was only nine and one, respectively (Table 5).

With regard to safety concerns, the incidence of interstitial pneumonitis was mildly higher than previously reported in the IPASS, EURTAC and LUX-LUNG6 studies (0%, 1%, and 0%, separately) [11,32,37,38]. One Japanese observational cohort with 3166 patients reported that the incidence of interstitial pneumonitis was 4% in the gefitinib group and 2.1% in the chemotherapy group [39]. The reported risks included: older age, poor performance status, smoking, recent lung cancer diagnosis, pre-existing chronic interstitial lung disease. However, in previous studies on TKI use in ICU lung cancer patients, adverse events were not mentioned clearly. In our study, two patients were diagnosed with interstitial pneumonitis. One was diagnosed by chest CT and erlotinib was then held. The other patient developed desaturation during treatment and their condition improved after steroid and antibiotics treatment with successful rechallenge of gefitinib. Although the diagnosis of TKI-related interstitial pneumonitis was not certain and the development of interstitial pneumonitis was not significantly related to survival in our study, the relatively higher incidence of possible interstitial pneumonitis in lung cancer patients with respiratory failure should be kept in mind. On the other hand, our study still revealed the benefit of the higher weaning rate in the patients receiving effective treatment. In addition, withholding EGFR TKI in cases without evidence of drug resistance during ICU admission could lead to disease recurrence. Previous studies on patients without any TKI-related toxicity found that 5–25% of the patients experienced disease flare-up after discontinuation of TKI [40,41,42]. According to the ASCO expert panel discussion in 2017, to stop the administration of EGFR TKI is reasonable only if there is apparent disease progression or intolerable side effects [43]. In the aspect of alternative treatment, immunotherapy also can cause severe immune-related adverse events in 20% of patients [44], and there was some evidence that revealed the limited efficacy of immunotherapy in patients with poor performance status [45]. Immune checkpoint inhibitors are also less effective in EGFR mutant lung cancer, precluding a useful application for these patients in the ICU setting [46]. According to our study results, it is worth administering EGFR-TKIs for patients who are detected as EGFR mutation while they are undergoing MV in ICU, and TKI should be withheld if there is any suspicion of TKI-related interstitial pneumonitis clinically.

There are several limitations in our study. First, this is a retrospective study performed in a single center. Though the case number is small, our study is the largest cohort of EGFR mutant lung cancer patients admitted to ICU with ventilator support. The EGFR mutation status and TKI-related outcomes were clearly documented and described. Second, many heterogeneities still existed in ICU patients despite multivariate adjustment. It is probably inapplicable to conduct a clinical trial to address the efficacy of EGFR TKI in the ICU setting; hence, our results might provide prognostic information for these patients in real world practice.

## 5. Conclusions

In conclusion, our study is currently the largest cohort to reveal the potential benefit of EGFR-TKIs use in NSCLC patients harboring a sensitizing EGFR mutation, especially the del19 subgroup, who were admitted to the ICU due to respiratory failure. Though sometimes difficult, obtaining a molecular profile using either tissue or liquid biopsy should be a mandatory approach to managing lung cancer patients with potential targetable driver mutations, even those in a critical status.

## Figures and Tables

**Table 1:** Demographic data and treatment outcome of EGFR-TKI-treated NSCLC patients who were admitted to the ICU and received mechanical ventilation.

EGFR Mutation (*n* = 35)		
Gender (Male/Female)	12 (34%)	23 (66%)
Age (median, range)	73 (67–79)	
Smokers (*n*, %)	8	23%
APACHE II score	25 (22–28)	
Stage IV (*n*, %)	34	97%
Interval between cancer diagnosis and ICU admission (days)	134 (6–546)	
Histology		
Adenocarcinoma (11 with histological subclassification) (*n*, %)	34	97%
Acinar (*n*, %)	6/11	54.5%
Papillary (*n*, %)	2/11	18.2%
Solid (*n*, %)	1/11	9.1%
Mucinous (*n*, %)	0/11	0%
Poorly differentiated (*n*, %)	2/11	18.2%
Sarcomatoid carcinoma (*n*, %)	1	3%
Comorbidity		
DM (*n*, %)	12	34%
HTN (*n*, %)	16	46%
COPD (*n*, %)	6	17%
CAD/HF (*n*, %)	3	9%
CKD (*n*, %)	3	9%
Reason for ICU admission		
Pneumonia (*n*, %)	28	80%
Shock (*n*, %)	6	17%
Cardiac-related (*n*, %)	1	3%
Neurological deficit (*n*, %)	3	9%
Operation (*n*, %)	1	3%
Type of EGFR mutation		
L858R (*n*, %)	15	43%
Deletion 19 (*n*, %)	14	40%
Uncommon (*n*, %)	6	17%
Metastatic site		
Lung-to-lung (*n*, %)	20	57%
Pleura (*n*, %)	25	71%
Pericardial effusion (*n*, %)	4	11%
Bone (*n*, %)	17	49%
Brain (*n*, %)	8	23%
Liver (*n*, %)	8	23%
EGFR-TKI treatment		
Gefitinib (*n*, %)	22	63%
Erlotinib (*n*, %)	11	31%
Afatinib (*n*, %)	1	3%
Osimertinib (*n*, %)	1	3%
Adverse events		
Interstitial pneumonitis (*n*, %)	2	6%
Diarrhea (*n*, %)	2	6%
Hepatitis (*n*, %)	1	3%
Skin toxicity (*n*, %)	4	11%
Outcome		
ICU 28-day-survival rate (*n*, %)	27	77%
Overall survival (days)	67 (31–320)	
Successful weaning from ventilator (*n*, %)	15	43%

Acronyms: APACHE II = Acute Physiologic Assessment and Chronic Health Evaluation (APACHE) II Scoring System, DM = diabetes mellitus, HTN = hypertension, COPD = chronic obstructive pulmonary disease, CAD/HF = coronary artery disease or heart failure, CKD = chronic kidney disease, EGFR = epidermal growth factor receptor, ICU = intensive care unit, TKI = tyrosine kinase inhibitor.

**Table 2:** Univariate and multivariate analysis of clinical factors associated with 28-day ICU survival.

	Univariate		Multivariate	
	OR (95% CI)		OR (95% CI)	*p* Value
Demographic factors				
Age	1.070 (0.993–1.153)	0.074	1.090 (0.990–1.199)	0.078
APACHE II	0.555 (0.117–2.634)	0.459	0.982 (0.834–1.157)	0.830
Gender (male vs. female)	1.054 (0.934–1.189)	0.397		
Brain metastasis	0.476 (0.087–2.593)	0.391		
Liver metastasis	1.051 (0.171–6.462)	0.958		
EGFR mutation (based on Deletion 19)				
L8585R	0.688 (0.124–3.786)	0.667		
Uncommon	0.375 (0.042–3.355)	0.380		
Comorbidity				
COPD	0.167 (0.023–1.232)	0.079	0.139 (0.011–1.764)	0.128
CAD/HF	0.667 (0.053–8.372)	0.753		
DM	0.294 (0.061–1.423)	0.128		
Reason for ICU admission				
Shock	0.167 (0.023–1.232)	0.079	0.017 (0.000–0.629)	0.027
Pneumonia	0.277 (0.029–2.637)	0.264		

Acronyms: APACHE II = Acute Physiologic Assessment and Chronic Health Evaluation (APACHE) II Scoring System, CAD/HF = coronary artery disease or heart failure, COPD = chronic obstructive pulmonary disease, DM = diabetes mellitus, EGFR = epidermal growth factor receptor, ICU = intensive care unit.

**Table 3:** Univariate and multivariate analysis of clinical factors associated with successful MV weaning.

	Univariate		Multivariate	
	OR (95% CI)		OR (95% CI)	*p* Value
Demographic factors				
Age	1.019 (0.920–1.046)	0.559	0.900 (0.791–1.026)	0.112
APACHE II	1.017 (0.915–1.130)	0.759	0.931 (0.777–1.116)	0.440
Gender (male vs. female)	1.875 (0.453–7.758)	0.386		
Brain metastasis	0.873 (0.172–4.429)	0.870		
Liver metastasis	0.873 (0.172–4.429)	0.870		
EGFR mutation (based on Deletion 19)				
L8585R	0.242 (0.052–1.133)	0.072	0.014 (0.000–0.450)	0.016
Uncommon	0.167 (0.015–1.879)	0.147	0.032 (0.001–1.358)	0.072
Comorbidity				
COPD	1.000 (0.145–6.907)	1.000		
CAD/HF	0.731 (0.033–3.284)	0.806		
DM	0.070 (0.008–0.635)	0.018	0.014 (0.000–0.416)	0.014
Reason for ICU admission				
Shock	0.327 (0.033–3.284)	0.342		
Pneumonia	2.014 (0.363–11.187)	0.423		

Acronyms: APACHE II = Acute Physiologic Assessment and Chronic Health Evaluation (APACHE) II Scoring System, CAD/HF = coronary artery disease or heart failure, COPD = chronic obstructive pulmonary disease, DM= diabetes mellitus, EGFR = epidermal growth factor receptor, ICU = intensive care unit.

**Table 4:** EGFR TKI treatment responses and weaning outcomes.

	EGFR Mutation (*n* = 35)	
	Weaning Success	Weaning Failure
CT image (*n* = 16)		
CR/PR	12	4
SD/PD	0	0
Chest radiography (*n* = 19)		
Improve	3	0
Stable/Deteriorate	0	16

Abbreviation: CR, complete response; CT, computed tomography; EGFR, epidermal growth factor receptor; PD, progression of disease; SD, stable disease.

**Table 5:** Summary of prior studies of EGFR-TKI use for lung cancer patients admitted to intensive care units.

Studies	Patient Population	Treatment	Outcomes
The present study	EGFR mutation: 35, EGFR wild-type: 28	All received EGFR-TKI	EGFR mutation vs. wild-type: 28-day ICU survival rate: 77% vs. 50%, *p* = 0.025 Median overall survival: 67 vs. 28 days, *p* = 0.01 Rate of weaning from MV: 43% vs. 25%, *p* = 0.14
Hsia et al. [6]	*n* = 83 (EGFR: 6) Respiratory failure	EGFR-TKI: 23 (6 with confirmed EGFR mutation)	Rate of weaning from MV: Standard care vs. EGFR-TKI: 18% vs. 22%, *p* = 0.81
Toffart AC et al. [35]	*n* = 14 (EGFR:5, ALK: 8, ROS1: 1) Respiratory failure (MV: 9, NIPPV: 4)	All received TKI	ICU survival rate 57% Median overall survival: 91 days Longer late survival versus histological control: HR 0.12, *p* = 0.002
Kerrigan et al. [17]	*n* = 9 (EGFR: 3, ALK: 3, ROS1: 1, MET: 1, unknown: 1) Respiratory failure (MV: 6, NIPPV: 3)	EGFR: Erlotinib: 3 ALK: Crizotinib: 1, Ceritinib: 1, erlotinib 1 ROS1: Crizotinib: 1 MET: Crizotinib: 1 Unknown: Erlotinib: 1	Rate of weaning from MV: 3 of 9 (33%) ICU mortality rate: 56%
Chen et al. [36]	*n* = 72 (EGFR was confirmed in only 1 case)	EGFR-TKI: 24 (1 with confirmed EGFR mutation)	ICU survival was better in patients receiving chemotherapy or EGFR-TKI vs. BSC (*p* = 0.011)

## Data Availability

All data are included in this manuscript.

## Supplementary Materials

The following are available online at www.mdpi.com/2227-9059/9/10/1416/s1, Figure S1: Survival and rate of successful weaning from mechanical ventilation of lung cancer patients receiving EGFR-TKIs in the ICU, Table S1: Demographic data of EGFR-TKI-treated NSCLC patients who were admitted to the ICU and received mechanical ventilation, Table S2: Treatment outcomes relevant to EGFR-TKIs.
