# Supplementary material for: Tyrosine Kinase Inhibitors Improved Survival of Critically Ill EGFR-Mutant Lung Cancer Patients Undergoing Mechanical Ventilation
Source: Biomedicines. 2021 Oct 8;9(10):1416. doi: 10.3390/biomedicines9101416 (PMC8533530; doi:10.3390/biomedicines9101416)
Supplement: Supplementary file 1 [file biomedicines-09-01416-s001.zip › biomedicines-1397359-supplementary.pdf]

# Supplementary Data

**Patient characteristics**

From 2010 to 2018, 176 patients admitted to the ICU with MV use and the diagnosis of NSCLC, and who were treated with EGFR-TKI, were enrolled. Fifty-one patients were excluded due to a lack of documentation of EGFR mutation status. Another 62 patients were excluded because of previous use of chemotherapy or other TKIs. Finally, 63 patients who received EGFR-TKIs as first-line therapy for lung cancer, and among whom 35 harbored a sensitizing EGFR mutation and 28 did not, were included. The median age of the patients was 73 years, 60% of the patients were female, and 68% were never-smokers. In terms of comorbidities, 11% of the patients had coronary artery disease or heart failure, and 21% had COPD. The major reason for ICU admission and MV use was pneumonia (87%). Most of the patients (61 of 63, 97%) were diagnosed with adenocarcinoma, and 2 patients had sarcomatoid carcinoma. All of the patients had stage 4 lung cancer, according to the American Joint Committee on Cancer 7th edition, and 19% had brain metastases. The mutation subtypes of the 35 (57%) patients who had a sensitizing EGFR mutation were as follows: L858R: 15 (42.8%), exon 19 deletion: 14 (40%), and uncommon mutation: 6 (17.1%). There was no significant difference between EGFR-mutant and wild-type cases, except patients with an EGFR mutation had a higher APACHE II score (EGFR wild-type versus EGFR-mutant: 20 versus 25 points, *p*-value = 0.002)

**Table S1.** Demographic data of EGFR-TKI-treated NSCLC patients who were admitted to the ICU and received mechanical ventilation.

| | **All (*n* = 63)** | | **EGFR Wild-type (*n* = 28)** | | **EGFR mutation (*n* = 35)** | | ***p* value** |
|---|---|---|---|---|---|---|---|
| Gender (Male) | 25 | 40% | 13 | 46% | 12 | 34% | 0.328 |
| Age (median, range) | 73 (63–80) | | 73 (56–84) | | 73 (67–79) | | 0.793 |
| Smokers (*n*, %) | 20 | 32% | 12 | 43% | 8 | 23% | 0.248 |
| APACHE II score | 23 (18–27) | | 20 (15–25) | | 25 (22–28) | | 0.002 |
| Stage IV (*n*, %) | 61 | 97% | 27 | 96% | 34 | 97% | 0.872 |
| Interval between cancer diagnosis and ICU admission (days) | 17 (0–376) | | 5 (0–45) | | 134 (6–546) | | 0.003 |
| **Histology** | | | | | | | |
| Adenocarcinoma (22 with histological subclassification) | 61 | 97% | 27 | 96% | 34 | 97% | 0.872 |
| Acinar | 10/22 | 45.5% | 4/10 | 40% | 6/12 | 50% | 0.658 |
| Papillary | 5/22 | 22.7% | 3/10 | 30% | 2/12 | 16.7% | 0.481 |
| Solid | 3/22 | 13.6% | 2/10 | 20% | 1/12 | 8.3% | 0.451 |
| Mucinous | 2/22 | 9.1%0 | 2/10 | 20% | 0/12 | 0% | 0.899 |
| Poorly-differentiated | 2/22 | 9.1% | 0/10 | 0% | 2/12 | 16.7% | 0.193 |
| Sarcomatoid carcinoma | 2 | 3% | 1 | 4% | 1 | 3% | 0.872 |
| **Comorbidity** | | | | | | | |
| DM | 20 | 32% | 8 | 29% | 12 | 34% | 0.628 |
| HTN | 25 | 40% | 9 | 32% | 16 | 46% | 0.274 |
| COPD | 13 | 21% | 7 | 25% | 6 | 17% | 0.444 |
| CAD/HF | 7 | 11% | 4 | 14% | 3 | 9% | 0.473 |
| CKD | 4 | 6% | 1 | 4% | 3 | 9% | 0.419 |
| **Reason for ICU admission** | | | | | | | |
| Pneumonia | 55 | 87% | 27 | 96% | 28 | 80% | 0.145 |
| Shock | 9 | 14% | 3 | 11% | 6 | 17% | 0.469 |
| Cardiac-related | 3 | 5% | 2 | 7% | 1 | 3% | 0.427 |
| Neurological deficit | 3 | 5% | 0 | 0% | 3 | 9% | 0.112 |
| Operation | 2 | 3% | 1 | 4% | 1 | 3% | 0.767 |
| **Type of EGFR mutation** | | | | | | | |

| | | | | | | | |
|---|---|---|---|---|---|---|---|
| L858R | | | | | 15 | 43% | |
| Deletion 19 | | | | | 14 | 40% | |
| Uncommon | | | | | 6 | 17% | |
| **Metastatic site** | | | | | | | |
| Lung-to-lung | 39 | 62% | 19 | 68% | 20 | 57% | 0.384 |
| Pleura | 41 | 65% | 16 | 57% | 25 | 71% | 0.237 |
| Pericardial effusion | 7 | 11% | 3 | 11% | 4 | 11% | 0.929 |
| Bone | 32 | 51% | 15 | 54% | 17 | 49% | 0.693 |
| Brain | 12 | 19% | 4 | 14% | 8 | 23% | 0.389 |
| Liver | 12 | 19% | 4 | 14% | 8 | 23% | 0.389 |

Acronyms: APACHE II= Acute Physiologic Assessment and Chronic Health Evaluation (APACHE) II Scoring System, DM= diabetes mellitus, HTN= hypertension, COPD= chronic obstructive pulmonary disease, CAD/HF= coronary artery disease or heart failure, CKD= chronic kidney disease

**Clinical Outcomes of all TKI-treated patients in ICU**

Most of the patients were treated with a first- or second-generation EGFR-TKI (gefitinib: 39, erlotinib: 22, and afatinib: 1). Only 1 patient received osimertinib treatment in the ICU. The median duration for the use of EGFR-TKIs in the ICU was 17 vs 8 days ($p = 0.001$) for patients with and without a sensitizing EGFR mutation. The 28-day ICU survival rate in patients with and without mutant EGFR was 77% and 50% ($p= 0.025$), respectively, with significant difference revealed by log rank test ($p= 0.015$). The median survival time for the whole cohort, EGFR-mutant, and EGFR wild-type patients was 52, 67, and 28 days, respectively. The Kaplan-Meier plotting of 28-day, 90-day, and overall survival is shown in Figures 2A, 2B and 2C. The log rank test showed significantly better 28-day, 90-day and overall survival with a $p$ value of 0.015, 0.010, and 0.001, respectively. In addition, 35% of the patients were successfully weaned from MV. The successful weaning rate was higher among EGFR-mutant cases, (EGFR-mutant versus wild-type: 43% versus 25%, $p = 0.14$); however, the Kaplan-Meier method did not show a significant difference in ventilator-free survival, with a log-rank $p$ value of 0.949 (Figure 2D). The clinical outcomes were summarized in Table S2.

**Table S2.** Treatment outcomes relevant to EGFR-TKIs.

| | All ($n$ = 63) | | Wild-type ($n$ = 28) | | EGFR + ($n$ = 35) | | $p$ value |
|---|---|---|---|---|---|---|---|
| **EGFR-TKI** | | | | | | | |
| Gefitinib | 39 | 62% | 17 | 61% | 22 | 63% | 0.001 |
| Erlotinib | 22 | 35% | 11 | 39% | 11 | 31% | |
| Afatinib | 1 | 2% | 0 | 0% | 1 | 3% | |
| Osimertinib | 1 | 1% | 0 | | 1 | 3% | |
| **EGFR-TKI treatment duration** | | | | | | | |
| Before ICU (days) | 0 (-6–129) | | -4 (-8–12) | | 52 (-4–487) | | 0.005 |
| During ICU (days) | 11 (7–21) | | 8 (4–11) | | 17 (10–27) | | 0.001 |
| **TKI adverse events** | | | | | | | |
| Interstitial pneumonitis ($n$, %) | 5 | 8% | 3 | 11% | 2 | 6% | 0.338 |
| Diarrhea ($n$, %) | 4 | 6% | 2 | 7% | 2 | 6% | 0.974 |
| Hepatitis ($n$, %) | 1 | 2% | 0 | 0% | 1 | 3% | 0.367 |
| Skin toxicity ($n$, %) | 4 | 6% | 0 | 0% | 4 | 11% | 0.181 |
| **Outcome** | | | | | | | |
| ICU 28-day-survival rate | 41 | 65% | 14 | 50% | 27 | 77% | 0.025 |
| Overall survival (days) | 52 (20–151) | | 28 (15–72) | | 67 (31–320) | | 0.005 |
| Successful weaning from ventilator ($n$, %) | 22 | 35% | 7 | 25% | 15 | 43% | 0.140 |

Acronyms: EGFR = epidermal growth factor receptor, ICU = intensive care unit, TKI = tyrosine kinase inhibitor

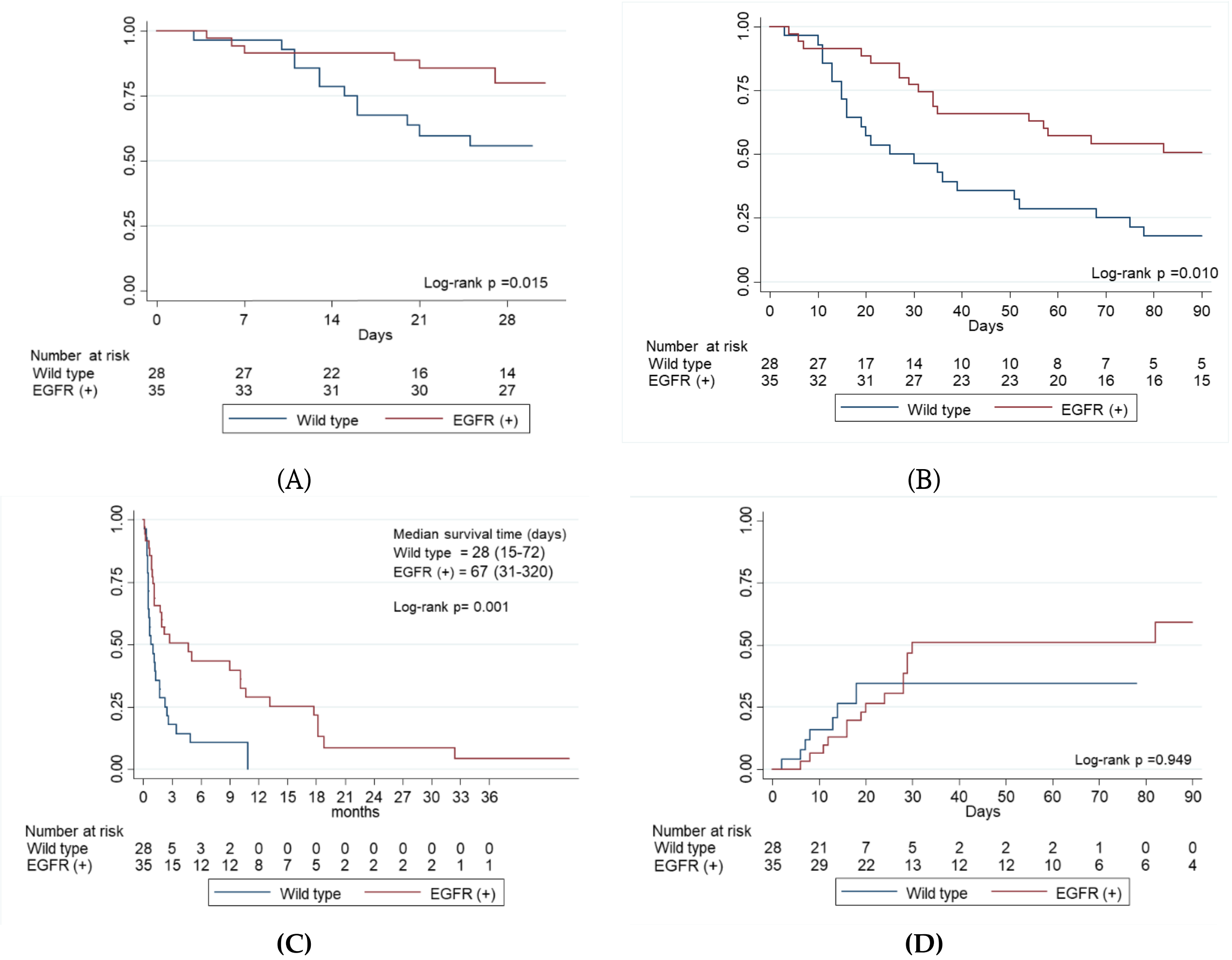


**Figure S1.** Survival and rate of successful weaning from mechanical ventilation of lung cancer patients receiving EGFR-TKIs in the ICU. (**A**) 28-day ICU survival. (**B**) 90-day survival. (**C**) Overall survival. (**D**) Cumulative incidence of patients with successful weaning from mechanical ventilators. Acronyms: EGFR = epidermal growth factor receptor, ICU= intensive care unit, TKI= tyrosine kinase inhibitor.
